# Effect of macrophage migration inhibitory factor on pulmonary vein arrhythmogenesis through late sodium current

**DOI:** 10.1093/europace/euac152

**Published:** 2022-09-03

**Authors:** Chye-Gen Chin, Yao-Chang Chen, Yung-Kuo Lin, Yen-Yu Lu, Wan-Li Cheng, Cheng-Chih Chung, Shih-Ann Chen, Yi-Jen Chen

**Affiliations:** Graduate Institute of Clinical Medicine, College of Medicine, Taipei Medical University, 250 Wu-Hsing Street, Taipei 11031, Taiwan; Division of Cardiovascular Medicine, Department of Internal Medicine, Wan Fang Hospital, Taipei Medical University, Taipei, Taiwan; Department of Biomedical Engineering, National Defense Medical Center, Taipei, Taiwan; Division of Cardiovascular Medicine, Department of Internal Medicine, Wan Fang Hospital, Taipei Medical University, Taipei, Taiwan; Division of Cardiology, Department of Internal Medicine, School of Medicine, College of Medicine, Taipei Medical University, Taipei, Taiwan; Division of Cardiology, Department of Internal Medicine, Sijhih Cathay General Hospital, New Taipei City, Taiwan; Division of Cardiovascular Surgery, Department of Surgery, Wan Fang Hospital, Taipei Medical University, Taipei, Taiwan; Division of Cardiovascular Medicine, Department of Internal Medicine, Wan Fang Hospital, Taipei Medical University, Taipei, Taiwan; Division of Cardiology, Department of Internal Medicine, School of Medicine, College of Medicine, Taipei Medical University, Taipei, Taiwan; Heart Rhythm Center and Division of Cardiology, Department of Medicine, Taipei Veterans General Hospital, Taipei, Taiwan; Division of Cardiology, Taichung Veterans General Hospital, Taichung, Taiwan; Graduate Institute of Clinical Medicine, College of Medicine, Taipei Medical University, 250 Wu-Hsing Street, Taipei 11031, Taiwan; Division of Cardiovascular Medicine, Department of Internal Medicine, Wan Fang Hospital, Taipei Medical University, Taipei, Taiwan

**Keywords:** Atrial fibrillation, Macrophage migration inhibitory factor, Pulmonary vein, Late sodium current, Calcium/calmodulin kinase II

## Abstract

**Aims:**

Macrophage migration inhibitory factor (MIF), a pleiotropic inflammatory cytokine, is highly expressed in patients with atrial fibrillation (AF). Inflammation increases the risk of AF and is primarily triggered by pulmonary vein (PV) arrhythmogenesis. This study investigated whether MIF can modulate the electrical activity of the PV and examined the underlying mechanisms of MIF.

**Methods and results:**

A conventional microelectrode, a whole-cell patch clamp, western blotting, and immunofluorescent confocal microscopy were used to investigate electrical activity, calcium (Ca^2+^) regulation, protein expression, ionic currents, and cytosolic reactive oxygen species (ROS) in rabbit PV tissue and isolated single cardiomyocytes with and without MIF incubation (100 ng/mL, treated for 6 h). The MIF (100 ng/mL)-treated PV tissue (*n* = 8) demonstrated a faster beating rate (1.8 ± 0.2 vs. 2.6 ± 0.1 Hz, *P* < 0.05), higher incidence of triggered activity (12.5 vs. 100%, *P* < 0.05), and premature atrial beat (0 vs. 100%, *P* < 0.05) than the control PV tissue (*n* = 8). Compared with the control PV cardiomyocytes, MIF-treated single PV cardiomyocytes had larger Ca^2+^ transients (0.6 ± 0.1 vs. 1.0 ± 0.1, Δ*F*/*F*_0_, *P* < 0.05), sarcoplasmic reticulum Ca^2+^ content (0.9 ± 0.20 vs. 1.7 ± 0.3 mM of cytosol, *P* < 0.05), and cytosolic ROS (146.8 ± 5.3 vs. 163.7 ± 3.8, Δ*F*/*F*_0_, *P* < 0.05). Moreover, MIF-treated PV cardiomyocytes exhibited larger late sodium currents (I_Na-Late_), L-type Ca^2+^ currents, and Na^+^/Ca^2+^ exchanger currents than the control PV cardiomyocytes. KN93 [a selective calcium/calmodulin-dependent protein kinase II (CaMKII) blocker, 1 μM], ranolazine (an I_Na-Late_ inhibitor, 10 μM), and *N*-(mercaptopropionyl) glycine (ROS inhibitor, 10 mM) reduced the beating rates and the incidence of triggered activity and premature captures in the MIF-treated PV tissue.

**Conclusion:**

Macrophage migration inhibitory factor increased PV arrhythmogenesis through Na^+^ and Ca^2+^ dysregulation through the ROS activation of CaMKII signalling, which may contribute to the genesis of AF during inflammation. Anti-CaMKII treatment may reverse PV arrhythmogenesis. Our results clearly reveal a key link between MIF and AF and offer a viable therapeutic target for AF treatment.

**What’s new?**

- Macrophage migration inhibitory factor may increase cytosolic reactive oxygen species, leading to activation of calcium/calmodulin-dependent protein kinase II (CaMKII). The calcium–calmodulin–CaMKII pathway can enhance L-type calcium current and facilitate late sodium current (I_Na-Late_), leading to triggering arrhythmia.
- KN93 (a selective CaMKII blocker) reduce pulmonary vein arrhythmogenesis, which regulated I_Na-Late_.

## Introduction

Atrial fibrillation (AF) is the most common type of sustained arrhythmia and causes substantial morbidity and mortality.^1^ Inflammation plays a major role in the pathogenesis of AF. Proinflammatory cytokine markers including C-reactive protein, tumour necrosis factor-α, interleukin-6, and macrophage migration inhibitory factor (MIF) are involved in the setting and prognosis of AF.^2^ Inflammation may contribute to atrial fibrosis and atrial structural remodelling, which may result in AF formation. However, the mechanisms underlying inflammation-mediated cardiac arrhythmogenesis remain poorly understood.

Macrophage migration inhibitory factor, a pleiotropic inflammatory cytokine, has been recognized as a mediator of numerous acute and chronic inflammatory diseases. Macrophage migration inhibitory factor controls the inflammatory ‘set point’ by regulating the release of other proinflammatory cytokines. Elevated MIF levels have been detected among patients with AF, indicating that MIF may play a key role in AF pathogenesis.^3^ However, research on the arrhythmogenic effects of MIF remains limited. Our previous study revealed that MIF increases atrial arrhythmogenesis through CD74 signalling by increasing the calcium (Ca^2+^) transient, sarcoplasmic reticulum (SR) Ca^2+^ content, Na^+^/Ca^2+^ exchanger (NCX) efflux rates, and level of Ca^2+^ leak.^4^ The pulmonary vein (PV) receives the most attention during AF initiation and maintenance.^5^ Macrophage migration inhibitory factor signalling activation was hypothesized to regulate PV arrhythmogenesis, contributing to AF genesis during inflammation.

Calcium/calmodulin-dependent protein kinase II (CaMKII) plays a vital role in atrial arrhythmia and PV arrhythmogenesis. The production of reactive oxygen species (ROS) greatly affects the progression of several inflammatory diseases. Reactive oxygen species are produced by cells that are involved in the host defence response. Furthermore, ROS promote endothelial dysfunction through the oxidation of crucial cellular signalling proteins such as tyrosine phosphatases.^6^ The overproduction of ROS and oxidation of CaMKII may lead to the abnormal up-regulation of the late sodium current (I_Na-Late_) and abnormal Ca^2+^ handling.^7^ This study investigated whether MIF modulates the electrophysiological characteristics of the PV by regulating ionic currents and Ca^2+^ homeostasis through the activation of CaMKII signalling.

## Methods

### Electropharmacological studies of pulmonary vein tissue preparations

All experiments were conducted in accordance with the *Guide for the Care and Use of Laboratory Animals*, published by the US National Research Council’s Institute for Laboratory Animal Research (animal permission number: LAC-2020-0271). All rabbits were anaesthetized with inhalational isoflurane (2.0–2.5%) from a precision vaporizer for 10 min. The adequacy of anaesthesia was determined by the lack of corneal reflexes or motor responses to painful stimuli induced with a scalpel tip. After the intravenous administration of heparin, a midline thoracotomy was performed and the heart and lungs were immediately excised. As described previously,^8^ PV tissues were dissected from the atria at the left atrium (LA)–PV junctions and from the lungs at the end of the PV myocardial sleeves. One end of the PV preparation (the PVs and LA–PV junctions) was pinned with needles at the bottom of a tissue bath, and the other end (distal PVs) was connected to a Grass FT03C force transducer (Grass Instruments, Quincy, MA, USA) with a silk thread. The PV tissues were perfused at a constant rate (3 mL/min) with Tyrode’s solution containing NaCl (137 mmol/L), KCl (4 mmol/L), NaHCO_3_ (15 mmol/L), NaH_2_PO_4_ (0.5 mmol/L), MgCl_2_ (0.5 mmol/L), CaCl_2_ (2.7 mmol/L), and glucose (11 mmol/L) and saturated with a gas mixture containing 97% O_2_ and 3% CO_2_. The PV tissues were treated with recombinant mouse MIF (100 ng/mL, 8 nM; Cat. No. 1978-MF/CF; R&D Systems) for 6 h. The electrophysiological effects were investigated with and without ranolazine (10 μM, the I_Na-Late_ inhibitor), KN93 (1 μM, a selective inhibitor of CaMKII), and *N*-(mercaptopropionyl) glycine[N-MPG; a hydroxyl (^•^OH) free-radical scavenger, 10 mM, a ROS inhibitor] superfusion.

The transmembrane action potentials (APs) of PV tissues were recorded using machine-pulled glass capillary microelectrodes filled with 3 mol/L KCl, which were connected to a World Precision Instruments Duo 773 Electrometer (Sarasota, FL, USA) under a tension of 150 mg. The temperature was maintained at 37°C, and the tissue preparations were equilibrated for 1 h before the electrophysiological and pharmacological evaluations. Electrical and mechanical events were displayed on a Gould 4072 Oscilloscope (Gould Electronics, Willoughby, OH, USA) and simultaneously recorded with a Gould TA11 Recorder, as described previously.^8^

### Isolation of single pulmonary vein cardiomyocytes

Single PV cardiomyocytes were isolated from the rabbits (2.0–3.0 kg).^8^ The isolated PV cardiomyocytes were incubated with recombinant mouse MIF (100 ng/mL, 8 nM; Cat. No. 1978-MF/CF; R&D Systems) for 6 h and then administered with ranolazine (10 μM, the I_Na-Late_ inhibitor), KN93 (1 μM, a selective inhibitor of CaMKII), and KN92 (1 μM, an inactive derivative of KN93) to investigate the electrophysiological effects.

A whole-cell patch clamp was used to record ionic currents and APs on isolated PVs with an Axopatch 1D amplifier (Axon Instruments, Foster City, CA, USA) at 35 ± 1°C. The ionic currents were recorded ∼3–5 min after rupture or perforation to avoid decay in ion channel activity. The micropipette resistance was 3–5 MΩ. At the beginning of each experiment, a small hyperpolarizing step was delivered from a holding potential of −50 mV to a test potential of −55 mV for 80 ms. The area under the capacitive current curve was divided by the applied voltage step to obtain the total cell capacitance. Typically, 60–80% of the series resistance was electronically compensated. A whole-cell patch clamp was used to record the I_Na-Late_, L-type Ca^2+^ current (I_Ca-L_), and the NCX current for the PV cardiomyocytes with and without MIF incubation (100 ng/mL) for 6 h in voltage-clamp mode. I_Na-Late_ was measured through a step-ramp protocol (−100 mV stepped to +20 mV for 100 ms, then ramped back to −100 mV over 100 ms) at room temperature using an external solution containing NaCl (130 mmol/L), CsCl (5 mmol/L), MgCl_2_ (1 mmol/L), CaCl_2_ (1 mmol/L), hydroxyethyl piperazine ethanesulphonic acid (HEPES, 10 mmol/L), and glucose (10 mmol/L) at pH 7.4 (adjusted with NaOH). Micropipettes were filled with a solution containing Na_2_ATP (4 mmol/L), CsCl (130 mmol/L), ethylene glycol tetraacetic acid (10 mmol/L), HEPES (5 mmol/L), and MgCl_2_ (1 mmol/L) at pH 7.2 (adjusted with NaOH). I_Na-Late_ was measured as the tetrodotoxin (30 μM)-sensitive portion of the traces obtained during voltage ramping back to 100 mV.

L-type Ca^2+^ current was measured as an inward current during depolarization from a holding potential of 50 mV to test potentials ranging from −40 to +60 mV in 10 mV steps for 300 ms at a frequency of 0.1 Hz. Measurements were taken using a perforated patch clamp with an external solution that contained tetraethylammonium chloride (137 mmol/L), CsCl (5.4 mmol/L), MgCl_2_ (0.5mmol/L), CaCl_2_ (1.8 mmol/L), HEPES (10 mmol/L), and glucose (10 mmol/L) at pH 7.4 (adjusted with CsOH). Micropipettes were filled with a solution containing CsCl (130 mmol/L), MgCl_2_ (1 mmol/L), MgATP (5 mmol/L), HEPES (10 mmol/L), Na-guanosine triphosphate (0.1 mmol/L), and Na_2_ phosphocreatine (5 mmol/L) at pH 7.2 (adjusted with CsOH). Steady-state inactivation of I_Ca-L_ was evaluated using a standard protocol consisting of a 300 ms pre-pulse and a 150 ms test pulse. The peak current elicited by the test pulse was divided by the maximal current and plotted as a function of the pre-pulse voltage. Data points were fitted with a Boltzmann function.

The NCX current was elicited by test pulses of between −100 and +100 mV from a holding potential of −40 mV for 300 ms at a frequency of 0.1 Hz. The amplitudes of the NCX current were measured as 10 mM nickel-sensitive currents. The external solution comprised NaCl (140 mM), CaCl_2_ (2 mM), MgCl_2_ (1 mM), HEPES (5 mM), and glucose (10 mM) at pH 7.4 and contained strophanthidin (10 μM), nitrendipine (10 μM), and niflumic acid (100 μM).

### Measurement of intracellular and sarcoplasmic reticulum Ca^2+^ content

Based on a previously described method, single PV cardiomyocytes were loaded with fluorescent Ca^2+^ (10 μM, fluo-3/AM) for 30 min at room temperature.^9^ Adopting the experimental design of Huang *et al*.,^10^ we loaded fluorescent Ca^2+^ (10 μmol/L) fluo-3/AM onto PV cardiomyocytes for 30 min at room temperature. After intracellular hydrolysis of fluo-3/AM for 30 min, excess extracellular dye was removed by changing the bath solution. Fluo-3 fluorescence was excited with the 488 nm line of an argon ion laser, and the emission was recorded at >515 nm. We repeatedly scanned cells at 2 ms intervals for line scan imaging (8-bit). Fluorescent imaging was performed with a laser scanning confocal microscope (Zeiss LSM 510; Carl Zeiss, Jena, Germany) and an inverted microscope (Axiovert 100; Carl Zeiss). To exclude variations in the fluorescent intensity caused by differences in the volume of injected dye and to correct for variations in dye concentrations, the fluorescent signals were calculated by normalizing the fluorescence (*F*) against the baseline fluorescence (*F*_0_), which yielded reliable information about transient intracellular Ca^2+^$\left(Ca_{i}^{2+}\right)$ changes from baseline values (*F*−*F*_0_, Δ*F*)/*F*_0_. The intracellular Ca^2+^ changes, including transient $Ca_{i}^{2+}$, peak systolic $Ca_{i}^{2+}$, and diastolic $Ca_{i}^{2+}$, were obtained during a 2 Hz field stimulation with 10 ms twice-threshold-strength square-wave pulses. When adding 20 mM caffeine after electrical stimulation at 2 Hz for at least 30 s, the estimated SR Ca^2+^ content as the total SR Ca^2+^ content was measured from the peak amplitude of the caffeine-induced $Ca_{i}^{2+}$ transients. After achieving a steady-state Ca^2+^ transients with the repeated pulses from −40 to 0 mV (1 Hz for 5 s), the SR Ca^2+^ content was estimated by integrating the NCX current following application of 20 mM of caffeine within 0.5 s during rest with the membrane potential clamped to −40 mV to cause SR Ca^2+^ release. The total SR Ca^2+^ content (expressed as mM of cytosol) was determined by use of the equation: SR Ca^2+^ content = [(1 + 0.12) (*C*_caff_/*F* × 1000)]/(*C*_m_ × 8.31 × 6.44), where *C*_m_ is the membrane capacitance, *F* is the Faraday’s number, cell surface to volume ratio is 6.44 pF/pL. To minimize cell motion during contraction, the scan line was positioned along the short axis (transversal scan) in the central region of the cell, avoiding the nucleus. A simplified estimate of SR Ca^2+^ fractional release was taken as Δ[Ca^2+^]*_i_* during a twitch divided by Δ[Ca^2+^]*_i_* during a caffeine application (Δ[Ca^2+^]twitch/Δ[Ca^2+^]caff), which provided an index of E–C coupling.

### Measurement of cytosolic reactive oxygen species

A cross-sectional area of each of the isolated single PV cardiomyocytes was imaged using a confocal laser scan microscope (Zeiss LSM 510), and the acquired images were processed using ImageJ measurement tools. We used CellROX Green (Life Technologies, Grand Island, NY, USA) to assess cytosolic ROS production in the freshly isolated control and MIF-treated PV cardiomyocytes at 1 Hz pacing. The measurements were performed using a laser-scanning confocal microscope (Zeiss LSM 510) and an inverted microscope (Axiovert 100) with a 63 × 1.25 numerical aperture oil immersion objective, as described previously.^7^ Freshly isolated PV cardiomyocytes were maintained in normal Tyrode’s solution (NaCl, 137 mM; KCl, 5.4 mM; CaCl_2_, 1.8 mM; MgCl_2_, 0.5 mM; HEPES, 10 mM) with 10 µM CellROX Green fluorescent dye. The CellROX Green dye was excited at 488 nm, and fluorescent signals were acquired at wavelengths of >505 nm in the XY mode of the confocal system. The acquired fluorescent images were analysed using Image-Pro Plus 6.0 and Sigma Plot 12 software, as described previously.^9^

### Statistical analysis

All continuous variables are expressed as the mean ± standard deviation. An unpaired Student’s *t*-test or one-way analysis of variance with a *post hoc* of Turkey was used to evaluate the time-dependent effect of MIF on PV electrical activity, and the differences in variables of ROS, intracellular Ca^2+^ homeostasis, contractility in PV preparations. A paired Student’s *t*-test was used to evaluate the effects of ranolazine, KN93, KN92, or N-MPG on electrical activity and ionic current in MIF-treated PV preparations. Comparisons between the different non-parametric variables were analysed using *χ*^2^ test with Fisher’s exact correction. A *P*-value of <0.05 indicated statistical significance.

## Results

### Effects of macrophage migration inhibitory factor on pulmonary vein electrical activity

**Figure 1:**
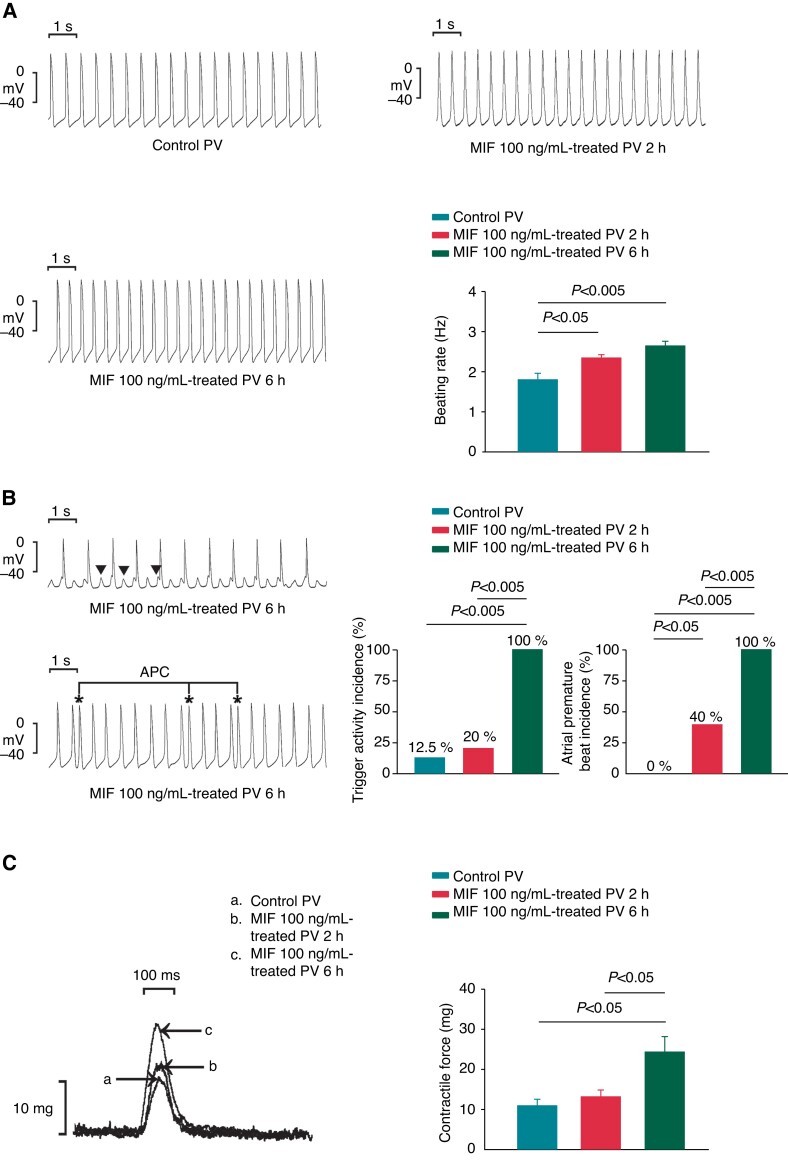
The spontaneous beating rates, incidence of trigger activity and premature atrial beat, PV contractility in control and MIF (100 ng/mL)-treated PV tissues. (*A*) The example and average data show that MIF (100 ng/mL)-treated PV tissues for 2 h (*n* = 5) or 6 h (*n* = 8) had faster beating rates than did control PV tissues (*n* = 8). (*B*) The upper panel illustrates the triggered activity (arrow), and the lower panel illustrates the atrial premature complexes. Tracings show that the MIF (100 ng/mL)-treated single PV tissues for 6 h (*n* = 8) had higher trigger activity and a higher incidence of premature atrial beat than did MIF (100 ng/mL)-treated single PV tissues for 2 h (*n* = 5) and control PV tissues (*n* = 8). (*C*) Superimposed tracings and average data indicate that MIF (100 ng/mL, *n* = 7)-treated PV tissues for 6 h had greater contractility than MIF (100 ng/mL)-treated single PV tissues for 2 h (*n* = 5) and control PV tissues (*n* = 7). MIF, macrophage migration inhibitory factor; N-MPG, *N*-(mercaptopropionyl) glycine; PV, pulmonary vein.

The MIF (100 ng/mL)-treated PV tissue preparations incubated for 2 or 6 h demonstrated a faster beating rate than the control PV tissue preparations (*Figure 1A*). However, the PVs treated with MIF for 6 h had a higher incidence of triggered activity and premature PV captures than the PVs treated with MIF for 2 h or control PV tissue preparations (*Figure 1B*). However, the 6 h MIF-treated PVs, 2 h MIF-treated PVs, and control PVs had similar maximum diastolic potential (−74.3 ± 1.9 vs. −73.9 ± 2.1 vs. −75.7 ± 1.9 mV). The 6 h MIF-treated PV tissue preparations also exhibited greater contractility than the control PVs and 2 h MIF-treated PV tissue preparations (*Figure 1C*).

### Effects of ranolazine, KN93, and *N*-(mercaptopropionyl) glycine on electrical activity on macrophage migration inhibitory factor–treated pulmonary vein preparations

**Figure 2:**
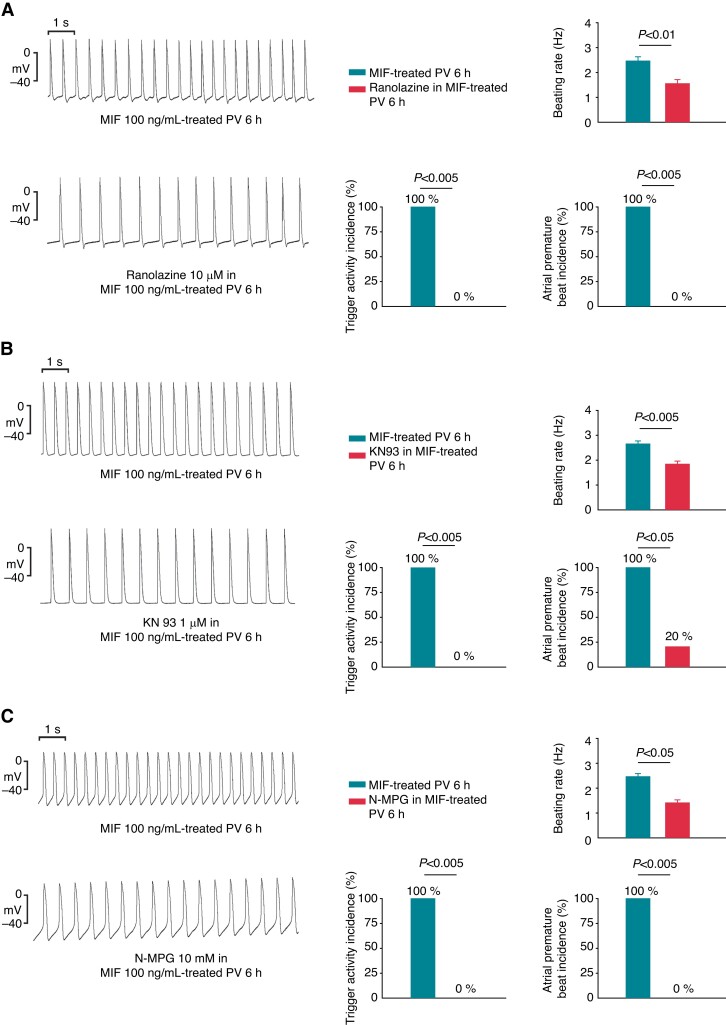
Effects of ranolazine (10 μM), KN93 (1 μM), and N-MPG (10 mM) on spontaneous beating rates, incidence of triggered activity, and premature atrial beat in MIF (100 ng/mL)-treated PV tissues for 6 h. (*A*) The left panel shows the tracings of MIF (100 ng/mL)-treated PV tissues before and after ranolazine (10 μM). The right panel shows that ranolazine (10 μM) reduced the beating rate, triggered activity, and premature capture in the MIF-treated PV tissues (*n* = 4). (*B*) The left panel shows the tracings of MIF (100 ng/mL)-treated PV tissues before and after KN93 (1 μM). The right panel shows that KN93 (1 μM) reduced the beating rate, triggered activity, and premature capture in the MIF-treated PV tissues (*n* = 5). (*C*) The left panel shows the tracings of MIF (100 ng/mL)-treated PV tissues before and after N-MPG (10 mM). The right panel shows that N-MPG (10 mM) reduced the beating rate, triggered activity, and premature capture in the MIF-treated PV tissues (*n* = 4). MIF, macrophage migration inhibitory factor; N-MPG, *N*-(mercaptopropionyl) glycine; PV, pulmonary vein.

Ranolazine (10 μM) reduced the beating rate of the 6 h MIF-treated PV tissue preparations. Ranolazine significantly decreased the incidence of triggered activity and premature capture in 6 h MIF-treated PV tissue preparations (*Figure 2A*). Similarly, KN93 (1 μM) and N-MPG (10 mM) reduced the beating rate and the incidence of triggered activity and premature captures in the 6 h MIF-treated PV tissue preparations (*Figure 2B* and *C*).

### Effects of macrophage migration inhibitory factor on I_Na-Late_, I_Ca-L_, and Na^+^/Ca^2+^ exchanger current of pulmonary vein cardiomyocytes

**Figure 3:**
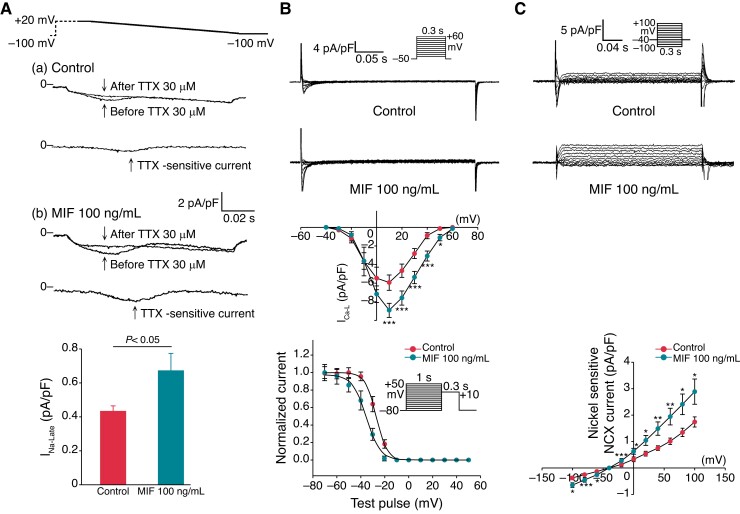
I_Na-Late,_ L-type calcium (I_Ca-L_), Na^+^/Ca^2+^ exchanger (NCX) in control, and MIF (100 ng/mL)-treated PV cardiomyocytes. (*A*) The current tracings and average data showed that MIF (100 ng/mL)-treated PV cardiomyocytes (*n* = 12) had higher I_Na-Late_ than control PV cardiomyocytes (*n* = 11). (*B*) Tracings, I–V relationship, and voltage dependence of inactivation and the recovery kinetics of I_Ca-L_ observed for control (*n* = 10) and MIF (100 ng/mL)-treated PV cardiomyocytes (*n* = 10). (*C*) Tracings and I–V relationship of NCX observed for control (*n* = 10) and MIF (100 ng/mL)-treated PV cardiomyocytes (*n* = 11). I_Ca-L_, I_Na-Late,_ L-type calcium; MIF, macrophage migration inhibitory factor; NCX, Na^+^/Ca^2+^ exchanger; TTX, tetrodotoxin; pA/pF, picoamperes per picofarad; *p < 0.05; **p < 0.01; ***p < 0.005.

The MIF (100 ng/mL)-treated single PV cardiomyocytes had a larger I_Na-Late_ (*Figure 3A*), larger I_Ca-L_ (*Figure 3B*), and larger reverse and forward NCX current (*Figure 3C*) than the control PV cardiomyocytes. Macrophage migration inhibitory factor–treated PV had a negative shift of the half-inactivation voltage (*V*_1/2_) of I_Ca-L_ (*Figure 3B*).

### Effects of KN93 and KN93 on I_Na-Late_, I_Ca-L_ and Na^+^/Ca^2+^ exchanger current of macrophage migration inhibitory factor–treated pulmonary vein cardiomyocytes

**Figure 4:**
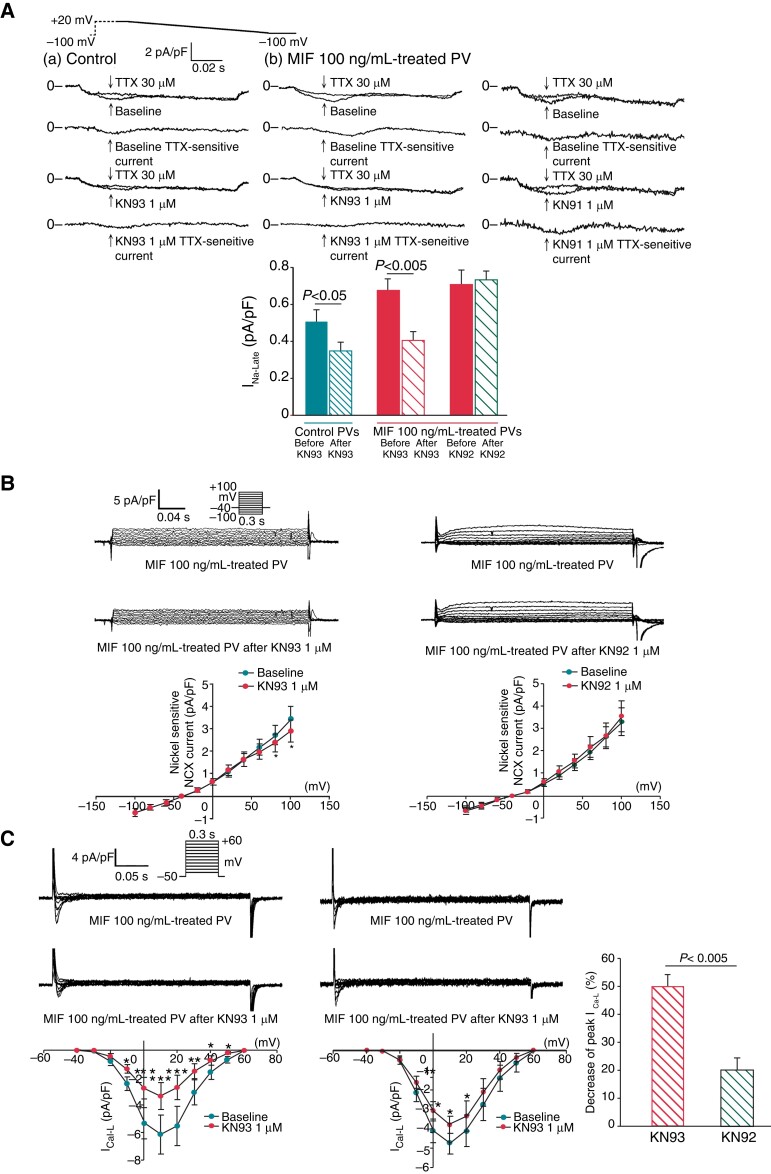
Effects of KN93, KN92 on I_Na-Late_, I_Ca-L_, and NCX on MIF (100 ng/ml)-treated PV cardiomyocytes. (*A*) Tracings and average data of I_Na-Late_ observed for control (*n* = 12) and MIF (100 ng/mL)-treated PV cardiomyocytes before and after KN93 (1 μM) (*n* = 11) or KN92 (1 μM) (*n* = 11) administration. KN93, but not KN92 reduced I_Na-Late._ (*B*) Tracings and average data of NCX observed for MIF (100 ng/mL)-treated PV cardiomyocytes before and after KN93 (1 μM) (*n* = 7) or KN92 (1 μM) (*n* = 7) administration. KN93, but not KN92 reduced reverse modes of NCX in the MIF-treated PV cardiomyocytes. (*C*) Tracings and average data of I_Ca-L_ observed for MIF (100 ng/mL)-treated PV cardiomyocytes before and after KN93 (1 μM) (*n* = 8) or KN92 (1 μM) (*n* = 6) administration. KN93 reduced percentage of peak I_Ca-L_ to a greater extent than KN92 in the MIF-treated PV cardiomyocytes. Peak I_Ca-L_ elicited from −50 to +60 mV. MIF, macrophage migration inhibitory factor; NCX, Na^+^/Ca^2+^ exchanger; PV, pulmonary vein.

The KN93 (1 μM), but not KN92, reduced I_Na-Late_ to a greater extent in the MIF-treated PV cardiomyocytes than in the control PV cardiomyocytes (–41.3 ± 11.3 vs. –31.2 ± 15.7%, *P* < 0.05; *Figure 4A*). Additionally, the control and MIF-treated PV cardiomyocytes had similar I_Na-Late_ levels when KN93 (1 μM) was used (*Figure 4A*). We found that KN93, but not KN92, significantly reduced reverse modes of NCX current (*Figure 4B*). Moreover, KN93 reduced I_Ca-L_ in the MIF-treated PV cardiomyocytes with a greater extent than KN92 (*Figure 4C*), suggesting that KN93 may have greater inhibition on I_Ca-L_ via its effect of CaMKII inhibition.

### Effects of macrophage migration inhibitory factor on intracellular reactive oxygen species and Ca^2+^ homeostasis

**Figure 5:**
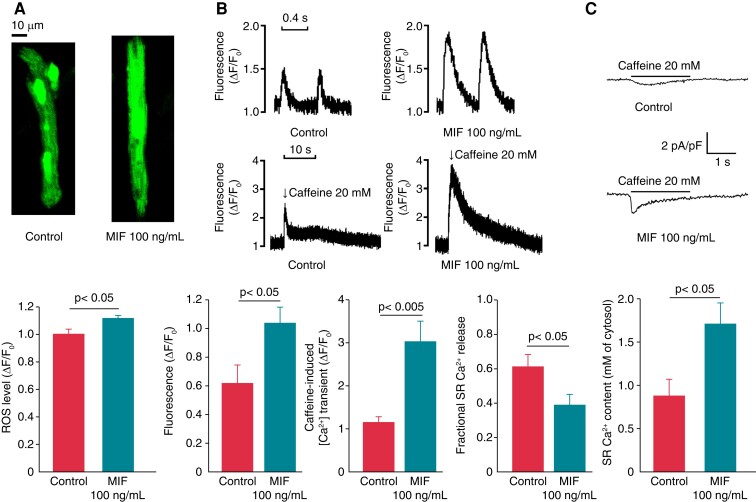
Cytosolic reactive oxygen species (ROS), intracellular calcium homeostasis, sarcoplasmic reticulum calcium stores of control, and MIF (100 ng/mL)-treated PV cardiomyocytes. (*A*) Example and average data of cytosolic levels of ROS in control (*n* = 16) and MIF (100 ng/mL)-treated (*n* = 16) PV cardiomyocytes. (*B*) Tracings and average data of calcium transients with or without caffeine and fractional SR Ca release in control (*n* = 10) and MIF (100 ng/mL)-treated (*n* = 10) PV cardiomyocytes. (*C*) Tracings and average data of caffeine-induced NCX and SR calcium content from the integration of NCX currents in control (*n* = 10) and MIF (100 ng/mL)-treated (*n* = 10) PV cardiomyocytes. MIF, macrophage migration inhibitory factor; ROS, reactive oxygen species; SR, sarcoplasmic reticulum; ROS, reactive oxygen species.

The MIF-treated PV cardiomyocytes had higher intracellular ROS than did the control PV cardiomyocytes (*Figure 5A*). We evaluated the effects of MIF on Ca^2+^ homeostasis and observed that the MIF (100 ng/mL)-treated PV cardiomyocytes had larger Ca^2+^ transients, lower fractional SR Ca release (*Figure 5B*) and greater SR Ca^2+^ content than the control PV cardiomyocytes (*Figure 5C*).

## Discussion

Inflammation is a key mechanism in AF genesis. However, the arrhythmogenic mechanisms of inflammation have not been fully elucidated. A previous study indicated that serum MIF concentrations are elevated in patients with AF.^3,11^ In this study, we investigated the effects of MIF on PV tissue and observed that MIF (100 ng/mL) increased PV tissue beating rate and the spontaneous triggered activity and led to a higher incidence of premature atrial beats, indicating that MIF may increase PV arrhythmogenesis, which may partially contribute to AF genesis. Moreover, the administration of ranolazine (10 μM) suppressed the arrhythmogenesis in the MIF-treated PV tissue preparations. These findings indicate that the modulation of I_Na-Late_ may contribute to MIF-enhanced PV arrhythmogenesis. Experiments on ionic currents have revealed that the MIF-treated PV cardiomyocytes had higher I_Ca-L_, NCX, and I_Na-Late_ densities than did the control PV cardiomyocytes. An increase in I_Na-Late_ and I_Ca-L_ can produce Ca^2+^ overload, leading to enhanced NCX activity in PV cardiomyocytes with greater arrhythmogenesis.^12^ Ca^2+^ image experiments demonstrated that the MIF-treated PV cardiomyocytes had a larger Ca^2+^ transient and SR Ca^2+^ content than the control cells, highlighting the critical role of MIF in cardiomyocyte Ca^2+^ homeostasis. Atrial fibrillation is associated with a decrease of I_Ca-L_ in atrial myocytes.^13^ In this study, we found that MIF increased I_Ca-L_ in PV cardiomyocytes (the main foci of AF triggers). Macrophage migration inhibitory factor led to an increase of I_Ca-L_ in PV cardiomyocytes, which may enhance PV beating rates and result in a higher PV arrhythmogenesis. These findings suggest that the initiation and the maintenance of AF may arise from different regulations of I_Ca-L_.

**Figure 6:**
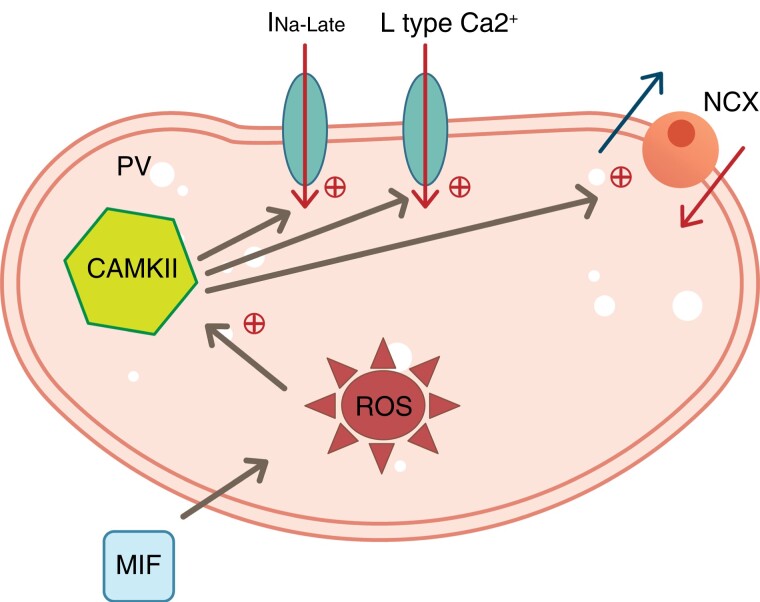
Proposed mechanism of MIF in the modulation of ion channels and intracellular calcium handling in PV cardiomyocytes. Macrophage migration inhibitory factor may increase cytosolic ROS, leading to increased intracellular calcium and the activation of CaMKII. The calcium–calmodulin–CaMKII pathway can enhance I_Ca-L_ and facilitate I_Na-Late_, leading to sodium–calcium overload and triggering ectopic beats. CaMKII, calcium/calmodulin-dependent protein kinase II; MIF, macrophage migration inhibitory factor; NCX, Na^+^/Ca^2+^ exchanger; PV, pulmonary vein; ROS, reactive oxygen species.

Reactive oxygen species can directly cause myocardial apoptosis and fibrosis. Increased levels of ROS, including superoxide and H_2_O_2_, have been associated with AF development.^14^ We determined that MIF induces ROS in the PV cardiomyocytes. Reactive oxygen species have been reported to be involved in cardiac electrical and structural remodelling.^15^ The molecular mechanisms underlying increased ROS production in AF and ROS-mediated downstream events have not been adequately determined. Reactive oxygen species produced by various sources, including nicotinamide adenine dinucleotide phosphate oxidase and mitochondria, have been noted to be involved in CaMKII activation. In this study, we observed that ROS-induced CaMKII activation may play a role in MIF-induced PV arrhythmogenesis. Moreover, KN93 or N-MPG (anti-oxidant) attenuated MIF-induced PV arrhythmogenesis, suggesting that ROS may activate CaMKII signalling. When KN93 was used, the control and MIF-treated PV cardiomyocytes had similar I_Na-Late_ levels. These findings indicate that MIF may increase I_Na-Late_ through the CaMKII pathway and thus cause abnormal Ca^2+^ homeostasis and Ca^2+^ regulation proteins in PV cells. The oxidation of the methionine residues of CaMKII serves as an indicator of increases in ROS level and is correlated with sustained kinase activity in the PV tissue.^16^ Similarly, previous studies have reported that ROS-activated CaMKII signalling may increase I_Na-L_ and arrhythmogenesis in PV cardiomyocytes.^17^ As illustrated in *Figure 6*, our study suggests that MIF may increase cytosolic ROS, leading to increased intracellular Ca^2+^ and the activation of CaMKII. The calcium–calmodulin–CaMKII pathway can enhance I_Ca-L_ and facilitate I_Na-Late_, leading to Na^+^/Ca^2+^ overload and triggering ectopic beats.^18^ A previous study reported that anti-MIF treatment for vascular smooth muscle homeostasis with 4,5-dihydro-3-(4-hydroxyphenyl)-5-isoxazoleacetic acid methyl ester may inhibit the aberrant transition of vascular smooth muscle cells in cardiovascular pathogenesis. Thus, anti-MIF treatment may be therapeutically beneficial in phenotype-related arterial remodelling.^19^ Moreover, inhibition of MIF/CD74 signalling reduced the extent of MIF-induced atrial arrhythmia in mice.^4^ A previous study indicated that MIF was related to electrical remodelling with AF, probably through falling I_Ca-L_ amplitudes and activating proto-oncogene tyrosine-protein kinase Src in the atrial myocytes.^20^ The current study also suggests that pharmacological intervention through direct MIF inhibition may modulate PV arrhythmogenesis. Accordingly, the agents targeting MIF may be used to treat AF.

## Conclusion

In conclusion, MIF has direct arrhythmogenic effects in PV cardiomyocytes through the activation of CaMKII signalling, dysregulation of Na^+^/Ca^2+^ homeostasis, and increment of ROS. Our results clearly uncovered an essential link between MIF and AF and offer a viable therapeutic target in AF.

## Data Availability

The data underlying this article will be shared on reasonable request to the corresponding author.
